# A Machine Learning Classification Model for Monitoring the Daily Physical Behaviour of Lower-Limb Amputees

**DOI:** 10.3390/s21227458

**Published:** 2021-11-10

**Authors:** Benjamin Griffiths, Laura Diment, Malcolm H. Granat

**Affiliations:** 1 School of Health and Society, University of Salford, Salford M5 4WT, UK; b.n.griffiths@salford.ac.uk; 2 People Powered Prosthetic Group, University of Southampton, Southampton SO17 1BJ, UK; laura.diment@soton.ac.uk

**Keywords:** classification, physical behaviour monitoring, machine learning, accelerometer, activity monitor, lower-limb amputee

## Abstract

There are currently limited data on how prosthetic devices are used to support lower-limb prosthesis users in their free-living environment. Possessing the ability to monitor a patient’s physical behaviour while using these devices would enhance our understanding of the impact of different prosthetic products. The current approaches for monitoring human physical behaviour use a single thigh or wrist-worn accelerometer, but in a lower-limb amputee population, we have the unique opportunity to embed a device within the prosthesis, eliminating compliance issues. This study aimed to develop a model capable of accurately classifying postures (sitting, standing, stepping, and lying) by using data from a single shank-worn accelerometer. Free-living posture data were collected from 14 anatomically intact participants and one amputee over three days. A thigh worn activity monitor collected labelled posture data, while a shank worn accelerometer collected 3-axis acceleration data. Postures and the corresponding shank accelerations were extracted in window lengths of 5–180 s and used to train several machine learning classifiers which were assessed by using stratified cross-validation. A random forest classifier with a 15 s window length provided the highest classification accuracy of 93% weighted average F-score and between 88 and 98% classification accuracy across all four posture classes, which is the best performance achieved to date with a shank-worn device. The results of this study show that data from a single shank-worn accelerometer with a machine learning classification model can be used to accurately identify postures that make up an individual’s daily physical behaviour. This opens up the possibility of embedding an accelerometer-based activity monitor into the shank component of a prosthesis to capture physical behaviour information in both above and below-knee amputees. The models and software used in this study have been made open source in order to overcome the current restrictions of applying activity monitoring methods to lower-limb prosthesis users.

## 1. Introduction

The World Health Organization (WHO) estimates that globally 100 M people need assistive products such as prosthetic devices, but up to 80–90% of this requirement is not currently being met [1]. Some of the reasons for this include an absence of policy, a lack of trained personnel, and the high cost of devices [1]. This issue is even more prominent in lower and middle-income countries, where the demographic of those in need of these devices is typically younger with increased physical working demands and where access to prosthetic services can be limited. Ensuring that the existing services provided are optimised is an important step in meeting these requirements. One way this can be achieved is by matching the correct prosthetic device to a user’s needs [2,3]. However, there are currently limited data on how these devices are actually used and how they support the functional ability of prosthesis users [3]. Traditionally, this information is captured by self-reporting from activity diaries or feedback from focus groups, but these subjective measures can be heavily influenced by social bias and patient recall [4,5]. Due to their small, unobtrusive size and low cost, body-worn sensors have become a commonly used tool for objectively measuring physical behaviour/activities in free-living environments.

Methods for objectively monitoring lower-limb amputees’ physical behaviours typically use step-count measurements to classify an individual’s activity level. Each person is then categorised into a physical function phenotype [3]. Although this method can easily establish an individual’s physical activity capacity, step count measurements do not provide detailed information on the user’s activities during these periods, providing limited information for assessing their physical behaviour. Measuring bodily postures such as lying, sitting, standing and movement would enable a more detailed analysis of their physical function, activity patterns and how their prosthesis impacts their lives [6]. For example, this information could enable researchers to analyse the duration of time spent in sedentary activity, which might indicate issues with an individual’s device such as poor prosthesis socket fit [7]. This would improve the understanding of a wearer’s prosthesis use and, therefore, the suitability of the device.

Only a few studies have investigated measuring the physical behaviours of lower-limb prosthesis users with a device capable of identifying postures, and most of these studies focused on validating the measurements against direct observations. van Rooij et al. [8] found that the overall average agreement between the thigh worn Activ8 activity monitor (2M Engineering Ltd., Valkenswaard, The Netherlands) and video observations was 97.3%. Similarly, Salih et al. [4] investigated the impact of wearing the activPAL device (PAL Technologies, Glasgow, UK) on the prosthesis socket and found a classification sensitivity of 90.5% for the non-amputated side and 86% for the amputated side in a population of unilateral lower-limb amputees. To date, only one study has looked at measuring lower-limb amputees’ physical behaviours in free-living. Bussmann et al. [9] found that individuals with a unilateral transtibial amputation caused by vascular disease are considerably less active than matched comparison subjects. Although this study provides useful information for clinicians, this research should be extended to different lower-limb amputee communities, providing a more detailed analysis.

There are several commercially available devices capable of measuring postures from raw acceleration data, such as activPAL and ActiGraph (ActiGraph LLC, Pensacola, FL, USA). These devices are worn on the thigh and provide accurate measurements in a range of populations [4,6,9]. The thigh is a favourable location to classify the postures that describe daily physical behaviour (lying, sitting, standing, and stepping) because each posture exhibits a unique gravitational-acceleration profile. This method was first developed by activPAL and has since been adopted by other device manufactures and used effectively in cohort studies [10]. However, for long-term monitoring and ease of use, prosthesis users have the unique opportunities to embed a device within their prosthesis. This would remove the requirement of taping a sensor to the skin, and the user would not need to remember to wear it, hopefully improving study compliance. The shank area of the prosthesis is a suitable location for both transtibial and transfemoral amputees and provides a stable interface to minimise movement artefact within the acceleration signal. However, a robust classification method is needed to ensure that the data are valid for this location.

Classifying amputee postures using a single shank worn accelerometer has only been explored by using threshold-based classifiers [6,11]. Redfield et al. [6] proposed a binary decision tree classifier based on accelerometer signal magnitude area for classifying postures, movement, and wear, achieving high classification accuracy (96.6%, SD = 3%). However, these data were collected by using a structured format, for short durations (<10 min), and were, therefore, unlikely to capture the posture variations present in unobserved free-living. This variation would reduce the accuracy of threshold-based classifiers, and this is supported by the findings of Kwon et al. [11], who found poorer classification accuracy (66.7%, SD = 6.0%) when using the same classifier on free-living data. Archer et al. [12] attempted to classify postures using both accelerometers and gyroscopes along with a decision tree and k-nearest neighbour classifier, achieving 95% sensitivity. However, the inclusion of a gyroscope sensor restricts the ability to collect data for longer durations due to increased power demands. Similarly, many studies have attempted to classify postures [13,14,15] and gait patterns [16,17,18,19,20] in both amputee and healthy populations using several sensors, but this does not transfer to a practical long-term monitoring solution due to the burden of wearing many sensors and the predicted decrease in compliance.

The aim of this study was to develop a model capable of accurately classifying lower-limb amputee postures by using data from a single shank-worn accelerometer. Many machine learning classifiers have proven capable of correctly recognising activities using different open access IMU datasets [21,22,23,24], including k-nearest neighbours, support vector machines, random forest classifiers, and neural networks. In this study, we explored the use of several machine learning classifiers with heuristic features to predict the postures that make up daily physical behaviour. Furthermore, one of the major drawbacks of research on activity classification is that the resulting models are often difficult to apply in practical monitoring applications as the data processing software is not available, and there is a lack of skills necessary to re-create the models. Therefore, the software and models developed as part of this study are available as open source to encourage their use in future physical behaviour monitoring research.

## 2. Materials and Methods

### 2.1. Data Collection

**Figure 1:**
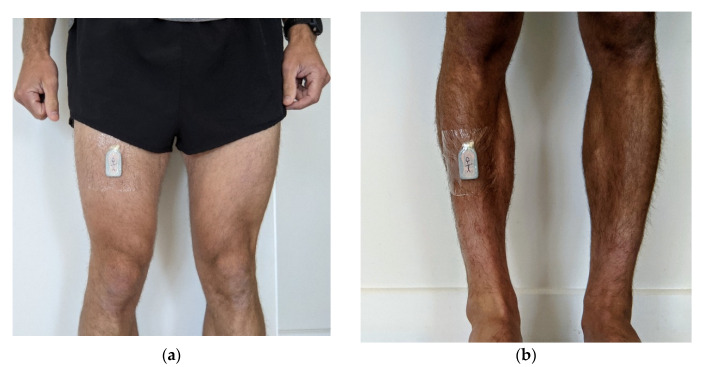
Experiment setup for activPAL PAL 3 in both positions: (**a**) thigh worn PAL3 for measuring postures; (**b**) shank worn PAL3 for measuring 3-axis accelerations.

Fifteen participants were recruited to participate in the study (male = 12; female = 3): 14 anatomically intact individuals and one double-transfemoral amputee. The mean participant age was 37.3 ± 9.4 years, height was 177.1 ± 7.1, and weight was 71.3 ± 7.8. The participants declared that they were fit and healthy with no comorbidities, and one amputee declared that they took part in regular exercise and frequently used their prostheses daily. Although this model is intended for use with lower-limb amputees, anatomically intact participants were selected due to the ease of recruitment and because their acceleration profiles in each posture are likely to be similar to amputees. The study was conducted according to the guidelines of the Declaration of Helsinki and approved by the Institutional Ethics Committee of the University of Salford (ID: 2068; Approved: 8 July 2021). Each participant consented to collect up to 7 days of free-living physical behaviour data during a typical week. The participants were requested to wear two commercially available 3-axis accelerometers (activPAL PAL3) on the anterior aspect of their thigh and shank, mid-way down each limb (Figure 1). The PAL3 was chosen for its validated posture classification algorithm within several populations and the ability to extract and analyse the raw accelerometer data. The device worn on the thigh was used to record the everyday postures of the wearer by using the in-built PAL3 classification algorithm (CREA. PAL Technologies. http://docs.palt.com/display/AL/CREA. Published 2020. Accessed on 2 May 2021) [25]. The device worn on the shank was used to capture the corresponding 3-axis shank accelerometer data at a sampling rate of 20 Hz. Both devices were fixed to the skin or prosthesis by using 3M Tegaderm tape and were only removed for bathing and swimming. If the participant removed their prosthesis or the monitor, the participant logged these periods in a diary, and these data were then removed during processing. The participants were asked to behave as they normally would throughout data collection, enabling the monitor to observe a broad range of free-living postures. At the end of the seven days, the participants returned the devices to the research team, where both the raw accelerometer data and classified events were extracted for analysis.

### 2.2. Data Processing

In order to create a classification training dataset, both raw data and event data were processed using the Python programming language (version 3.7.4). The PAL3 event data were exported as posture codes, where each posture is classified by using its actual duration and not in windows with fixed durations. Each code represented one of the following posture classes predicted by the PAL3 software: sedentary/sitting, standing, stepping, cycling, primary lying, secondary lying, non-wear, and travelling. These data were then transformed into six different datasets of fixed windows ranging from 5 s to 3 min (5, 15, 30, 60, 120, and 180 s). Windowing was chosen because it enabled the analysis of different classification algorithms, while multiple window lengths were chosen to explore the impact of window length on prediction accuracy. Each window contained both the thigh PAL3 assigned posture class and the corresponding raw shank accelerometer data. Windows that overlapped multiple postures were removed from the dataset to ensure good class separation for developing the model. Any non-wear and travelling posture classes were removed from the dataset, while related posture classes were grouped into one combined class in order to reduce the complexity of the model. For example, cycling was combined with stepping, and primary and secondary lying were combined. The data were extracted by using a 50% overlapped sliding window to capture more unique posture data for training the models.

The 3-axis accelerometer data were used to calculate 100 time and frequency domain features for each window. Each accelerometer axis was first filtered using a 4th order low-pass Butterworth filter with a cut-off frequency of 5 Hz [26] and then combined to create a vector magnitude (VM) signal. The resulting filtered X, Y, Z, and VM acceleration signals were used to calculate the features. For the full list of features, refer to Table 1; each of these features were calculated for the four signals. These features were chosen from previous activity classification research [12,27,28]. Finally, all data were combined to create a single dataset for each window length.

### 2.3. Classifier Development

The features contained within each dataset were analysed to understand which feature was the most useful for separating the posture classes. They were first scaled to their minimum and maximum sample; thus, each value fell between 0 and 1, and then any strongly correlated features (Pearson’s correlation >0.8) and quasi-constant features (variance <0.01) were removed from the dataset. Typically, this left 30–35 features depending on the window length.

Each classifier was trained by using the Python Scikit-Lean library (version 0.22). The classifiers chosen for analysis were K Nearest Neighbour (KNN), Linear Discriminant Analysis (LDA), Support Vector Machine (SVM), Random Forrest (RF), Extra Trees (ET), Logistic Regression (LR), Naive Bayes (NB), and Quadratic Discriminant Analysis (QDA). These algorithms were chosen because they required minimal pre-processing and performed well on similar classification problems [29,30,31]. Each classifier’s hyperparameters were tuned on a stratified subset of the dataset (10%) using an 80% train and 20% test data setup. This was performed on each of the windowed datasets in order to find the best performing version of each. During the training, each dataset’s classes were balanced to prevent classification bias by having an overwhelming amount of one specific class, which was assumed to be highly likely based on the typical duration each participant spent lying down and sitting compared to standing and stepping.

The datasets and classifiers were assessed by using a 10-fold stratified cross-validation, where 1/10th of the data was systematically removed from the dataset for testing while the remaining data were used for training. This is repeated until all the data had been tested. Stratification enabled consistent class balance across the training and testing datasets. F-scores were used to assess the accuracy of each model and were calculated by using both the precision and recall of each cross-validation pass. Furthermore, average confusion matrices from the 10-fold cross-validation were created to describe how the postures were misclassified.

## 3. Results

The amount of data collected from each participant ranged from 3 days to 7, so only the first 3 days of data were used to ensure each individual contributed equally to the dataset. This resulted in datasets with between 787,658 and 14,433 samples for the 5 s and 180 s windows, respectively. As predicted, there were large discrepancies between the quantity of each posture class instance based on each participant’s physical behaviour pattern. After balancing these classes by removing random samples from the more prominent classes, the resulting datasets contained between 85,835 and 685 samples for the 5 s and 180 s windows.

The feature selection stage of the processing found an average of 69 correlated and quasi-constant features within each window length’s dataset. There was a trend for more correlated and quasi-constant features as window length increased. The most popular prediction features included each acceleration signal’s mean value, standard deviation, max and min values, and spectral peak-frequencies. Using this subset of features reduced the computation time needed for each dataset and had a notable impact on the model’s development and inference time.

**Figure 2:**
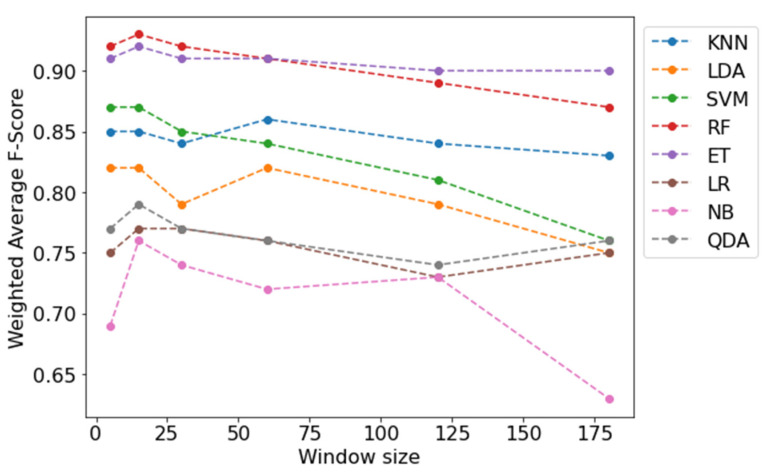
F-scores for each algorithm across all window lengths.

The F-scores for each model and window length are shown in Figure 2. For every model other than the KNN, the 15 s window length achieved the highest F-scores, while there was a trend of poorer performance as the window length increased, with the 120 and 180 s windows performing the worst. The RF classification model achieved the best individual result of 93% compared to the poorest result of 63% from NB with 180 s window length. On average, the RF and ET algorithms performed the best with 91% average F-scores across all window lengths, followed by KNN (86%), SVM (83%), LDA (80%), QDA (77%), LR (76%), and NB (71%).

The F-scores for the different posture classes and weighted average F-scores for each model are shown in Table 2. To reduce the complexity, only the best performing 15 s window length dataset is presented. Sitting was consistently the most misclassified posture with an average F-score of 69% across all classifiers. Meanwhile, the average F-scores for standing events were slightly higher (79%), but lying (91%) and stepping (96%) events consistently achieved high F-scores across all classifiers.

**Figure 3:**
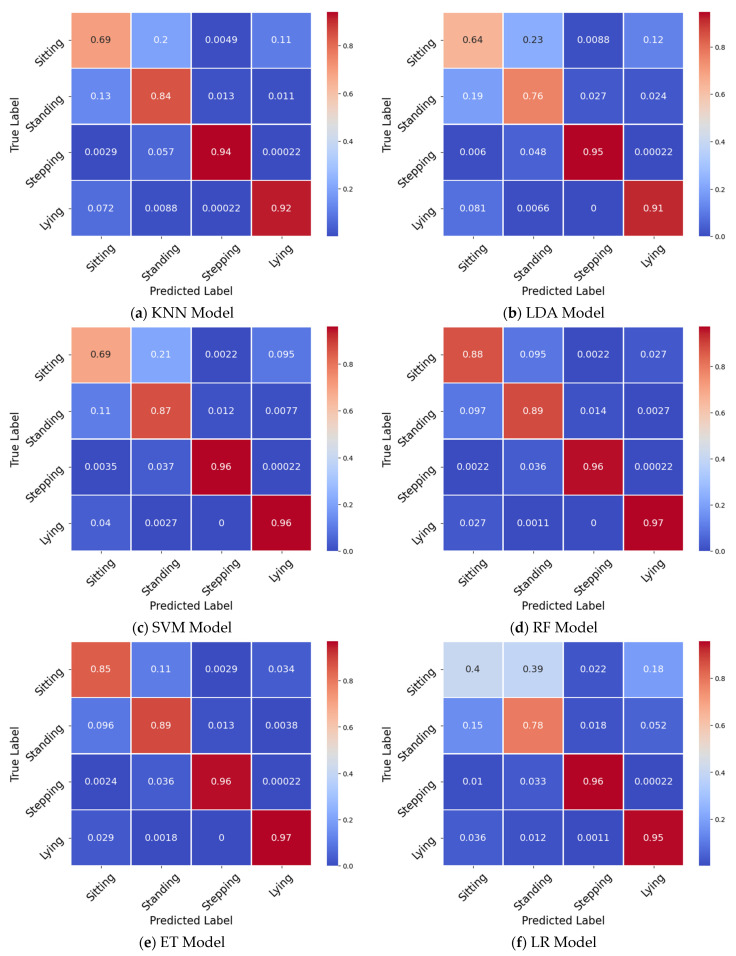

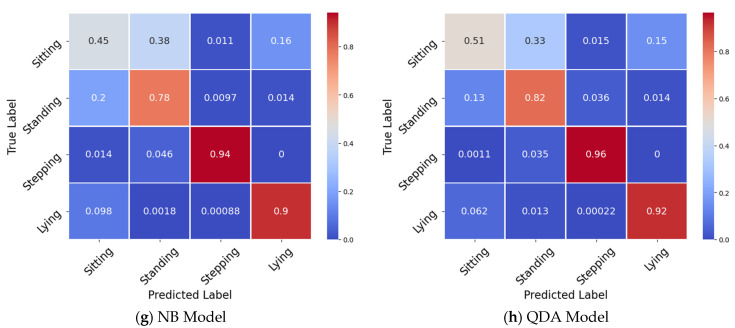
Confusion matrices for each classifier utilizing a 15 s window length: (**a**) KNN, (**b**) LDA, (**c**) SVM, (**d**) RF, (**e**) ET, (**f**) LR, (**g**) NB, and (**h**) QDA.

Confusion matrices show the percentage of misclassified postures for each class (Figure 3). Across all the classifiers, misclassified sitting postures were more likely to be classified as standing postures (11–39%). Similarly, misclassified standing postures were more likely classified as sitting postures (1–20%). Although lying postures were consistently classified correctly, those few misclassifications were more likely to be classified as either of the other static postures (<1–10%), whereas stepping postures were most likely misclassified as standing (3–6%).

**Figure 4:**
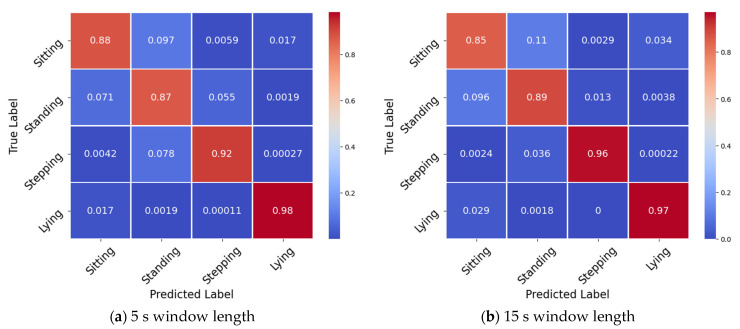

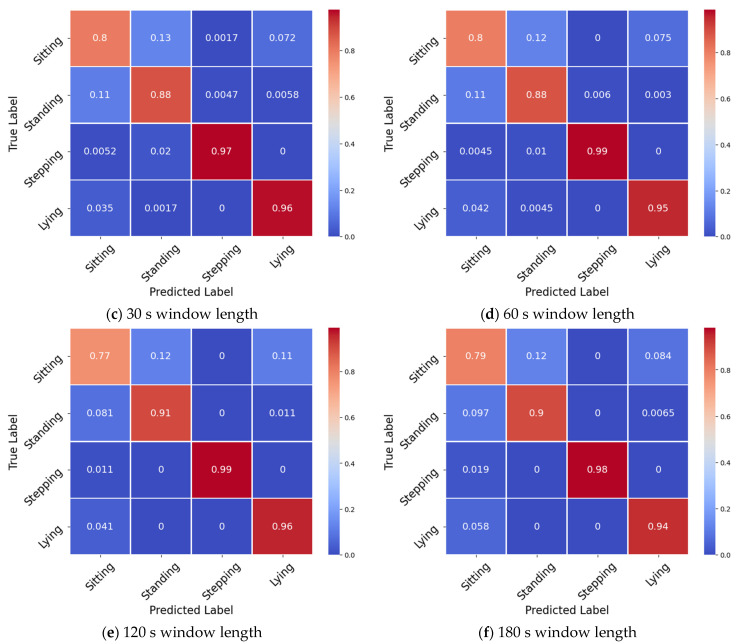
Confusion matrices for the RF classifier across the different window lengths: (**a**) 5, (**b**) 15, (**c**) 30, (**d**) 60, and (**e**) 120, (**f**) 180.

Confusion matrices for the different window lengths for the best performing RF classifier are shown in Figure 4. As the window length increased, there was a trend of increased misclassification when trying to predict sitting postures (12% 5 s window length and 21% 180 s window length) and, conversely, a small increase in performance at classifying standing postures (13% 5 s window length and 10% 180 s window length). Increasing the window length from 5 s to 15 s improved stepping posture classification by 4%. This gain in performance slowly grew to 99% accuracy for the 120 s window length before dropping down again at 180 s (98%). Lying postures remained largely unaffected, only losing a small amount of performance as the window length increased (4%).

## 4. Discussion

This study shows that data from a single shank worn accelerometer with a machine learning classification model can be used to accurately identify the postures that make up an individual’s daily physical behaviour. These models, trained and evaluated using free-living data, achieved F-scores greater than 90%. The RF classifier offered the best performance of 93% weighted average F-score and between 88 and 98% classification accuracy across all four posture classes; this is the best performance achieved to-date by a shank-worn device. These models achieved results that enable the use of a shank-worn system to analyse free-living physical behaviours, which is traditionally performed by using thigh and hip worn accelerometers. Therefore, this offers an opportunity for a more discrete and unobtrusive monitoring system that is applicable to all lower limb amputees.

The performance achieved by these models highlights the advantage of developing a classifier using machine learning methods as opposed to threshold-based classifiers. Previous research has shown that the accuracy of threshold-based classifiers is reduced by up to 40% when tested on free-living data compared to previously presented results on laboratory data [11]. This has been attributed to the variation in the execution of different postures typically found in free-living conditions, as opposed to the strict and considered movements performed in a laboratory. The limited flexibility of threshold-based classifiers means that they cannot account for these variations in posture execution. In contrast, machine learning models have more complex classification methods and can identify these differences. Furthermore, this study’s method of data collection enabled a wide range of examples of the same posture to be collected over multiple days, capturing the variations in these postures. The results of this study are more comparable to the results presented by Redfield et al. [6] who tested a threshold-based classifier on laboratory data (96.6% vs. 93%), showing the improved performance of machine learning models when applied to free-living data.

The classification performance of the models developed in this study is consistent with the findings of previous studies attempting to classify other postures and activities by using machine learning models. For example, Ernes at al. [31] used a combined decision tree and neural network classification architecture to classify several postures (lying, sitting, standing, and walking) and activities, achieving an accuracy of 89% when using laboratory and free-living data. Interestingly, they also projected a 17% reduction in classification accuracy when training their model on only laboratory data and testing it on free-living data, showing that the reduction in accuracy when training a model with laboratory data is less than that of threshold-based classifiers. Bastian et al. [30] attempted to classify a similar posture set of lying, slouching, sitting, standing, walking, running, and cycling by using a hip worn accelerometer and Bayesian model. They achieved high specificity (>90%) when classifying both static and dynamic postures using free-living data, with significant improvements over models trained using laboratory data. Similarly, Ahmadi et al. [29] achieved F-scores of 86.4% when assessing the playtime activities of children using hip and wrist worn accelerometers with a RF classifier. Although the models in these studies were developed using different accelerometers, wear locations, number of sensors, and population types, they achieved relatively similar accuracies. This similarity shows the appropriateness of machine learning models when attempting to classify postures and activities in free-living, while highlighting the need for varied examples of training data collected in the test environments.

This study showed that window length has a significant impact on a model’s performance. A range of durations was tested to understand the effect of increasing and decreasing window length on average classification accuracy and each posture. It was hypothesised that decreasing the window length would enable more data to be collected by reducing the chance of windows spanning multiple postures, but larger windows would capture more information on the movement in order to help differentiate between the postures. Specifically, the challenge was differentiating between sitting and standing postures as the accelerometer is orientated along the same axis, unlike lying, and there are similar amounts of movement, unlike stepping. However, the results show that a 15 s window provides the best performance across all but one of the models. Furthermore, increasing the window length decreased the performance of each algorithm by 2–16%, which was mainly caused by the misclassification between sitting and standing postures. It is likely that the longer duration windows enabled more variable movements to take place in these postures, introducing noise to the features and reducing their separability. Future studies should focus on feature engineering to improve the ability to differentiate between sitting and standing before fully assessing the impact of window length. However, for this feature set, a 15 s window length is optimal.

Model development is an important part of creating a posture/activity classifier, and this encompasses four key steps: (1) feature selection, (2) feature engineering, (3) model selection, and (4) hyperparameter tunning. This study explored the use of a merged set of features from previous research on similar activity classification problems [12,27,28], which included time domain and frequency domain features. These features were subjected to a typical feature engineering workflow including normalisation, before removing features that had low variance or were highly correlated. However, this process could be further investigated by using additional features and feature engineering methods such as using features derived from discrete wavelet transforms or adjacent windows [29,32]. This could enhance the performance of the classifiers presented here by introducing temporal information to the model, which is relevant to this application as posture changes are event related, e.g., you must stand from sitting before you can start walking. Several models were explored in this study with some performing far better than others. The RF and ET classifiers are both types of ensemble algorithms and, unsurprisingly, achieved similar high performance. The SVM and KNN classifiers also achieved high F-scores (>85%) and have previously been applied to other classification problems with similar performance [29,30,31], proving their validity for solving these problems. In addition to this, further studies should investigate the use of deep learning in order to automatically develop features, removing the need for the feature generation and engineering stages of this process. This could have a significant impact on performance by optimising feature development and introducing temporal features by using recursive neural networks. The tuning of the model hyperparameters can have a small impact on performance, and within this paper, the parameters that make up each model were explored to find the best performance on a train–test split dataset. This process should be explored when developing new classifiers as it can be highly dependent on the dataset under investigation.

The data collection method presented in this paper introduces a novel method of automatically collecting labelled, free-living posture data for developing posture/activity classifiers. Using the PAL3′s existing classification algorithm enables constant and long-term data collection from a validated device along with corresponding 3-axis acceleration data. This makes it much easier to collect free-living posture data as opposed to labelling video data or conducting manual observations, which are common methods used in similar studies [6,12,29]. As this method requires no additional work, it is likely to reduce the chance of human errors that are typically present with repetitive tasks. This method also captures large volumes of data from multiple participants, which is important for developing machine learning models that require large quantities of training data. However, previous research has shown that the PAL3 posture classification algorithm achieves 90% accuracy when classifying amputee postures in free-living. This could introduce inherent errors within the developed classifiers and should be considered when drawing conclusions from these results. Future studies should assess models developed using this data collection method alongside direct observations in order to fully understand the impact of these errors. This study only used windows containing single postures and not windows that spanned multiple postures, and it is likely that the performance will be different when testing these classifiers on mixed datasets, although Ahmadi et al. [29] found an increase in performance when introducing mixed windows, which was attributed to an increase in the quantity of training data for their models. Future studies should look to investigate the impact of mixed windows on the accuracy of these models. Finally, this method extracted data by using a 50% overlapping window to collect more unique data for model development. However, there is the potential to alter the amount of overlap which could increase the quantity of data collected and aid development. Future investigations should experiment with increasing and decreasing the window overlap to understand how this impacts model development.

The results of this study could have a significant impact on the evaluation of lower limb amputees’ free-living physical behaviours and their prosthesis use. Technical limitations, such as the cost of sensors, lack of clear methods, and software that is not simple to use, mean that using sensors to monitor amputees’ physical behaviours and their prosthesis is not standard clinical practice [3]. This issue is more prominent in lower-income and middle-income countries where products are often poorly suited to their environment and the user’s needs. This study presents a method for accurately measuring daily postures by using a shank worn accelerometer that can be easily housed within all lower-limb prosthetic devices. These measures offer the opportunity for an extensive evaluation of a wearer’s physical behaviours and could help understand the broader context of those behaviours. Furthermore, the software and models developed as part of this study are open access in order to overcome some of the limitations that prevent these monitoring methods from being applied. However, the main limitation of this study is that the models were developed by using data from a single amputee and many anatomically intact individuals. Although an initial evaluation of the data showed similar variances between feature characteristics of this amputee and the other participants, it is not possible to say that the model accuracy presented in this paper will directly translate to an amputee population. It is also likely that different prosthesis types, such as transfemoral or transtibial, and familiarity with the prosthesis could impact the shank acceleration profiles and model accuracy. Future studies should investigate the performance of these models on different lower-limb amputee populations and compare the features calculated from shank-accelerations to a non-amputee population.

## 5. Conclusions

This research developed a model capable of accurately classifying lower-limb amputee postures by using data from a single shank-worn accelerometer. The RF classifier provided the highest average classification accuracy of 93% across all four posture classes, and a 15 s window length appears to be the most appropriate for classifying postures by using machine learning algorithms. This method offers the opportunity to embed an accelerometer-based activity monitor into the shank component of a prosthesis to capture physical behaviour information in both above and below-knee amputees. This could help us understand how prosthetic devices are actually used by amputees, how they support their functional abilities, and the impact of changes to prosthesis design. The models and software (see Supplementary Materials) used in this study have been made open source in order to overcome the current restrictions on applying activity monitoring methods to lower-limb prosthesis users and to enable their use in future research.

## Figures and Tables

**Table 1:** Table of calculated time domain and frequency domain features for each window.

Time Domain Features	Frequency Domain Features
Mean	1st Autocorrelation Coefficient
Standard Deviation	Power of First 6 Spectral Peaks
Mean Absolute Deviation	Frequency of First Spectral Peaks
Maximum Sample Value	Total Power in 4 Adjacent Frequency Bands
Minimum Sample Value	
Signal Magnitude Area	
Signal Energy	
Interquartile Range	

Features were calculated for all 4 signals (X, Y, Z, and VM).

**Table 2:** Table of F-scores for each posture class and weighted average F-scores for each model with a 15 s window length.

Algorithm	Sitting	Standing	Stepping	Lying	Weighted Ave F-Score
KNN	0.74	0.81	0.96	0.9	0.85
LDA	0.68	0.75	0.96	0.89	0.82
SVM	0.76	0.83	0.97	0.93	0.87
RF	0.88	0.88	0.97	0.98	0.93
ET	0.87	0.87	0.97	0.97	0.92
LR	0.51	0.72	0.96	0.88	0.77
NB	0.51	0.71	0.96	0.87	0.76
QDA	0.59	0.74	0.96	0.88	0.79

## Data Availability

The data presented in this study are available on request from the corresponding author. The data are not publicly available due to privacy.

## Supplementary Materials

The final models and processing software (Python) are available at the following repository: https://github.com/Ben-Jamin-Griff/ProsNet (accessed on 8 November 2021).
